# Immune-mediated multisystem injury associated with monoclonal gammopathy of clinical significance: a case report

**DOI:** 10.3389/fimmu.2026.1833299

**Published:** 2026-05-08

**Authors:** Xianghong Jin, Xinmin Duan, Runfeng Zhang, Yuxin Sun, Min Qian, Xingyu Li, Donglai Ma, Xiao Li, Xiaoqian Zhang, Min Shen, Xuejun Zeng, Na Xu, Junling Zhuang

**Affiliations:** 1 Department of Rare Diseases, Peking Union Medical College Hospital, Chinese Academy of Medical Sciences & Peking Union Medical College, Beijing, China; 2 Department of Internal Medicine, Peking Union Medical College Hospital, Chinese Academy of Medical Sciences & Peking Union Medical College, Beijing, China; 3 Anaesthesiology, National Cancer Center/National Clinical Research Center for Cancer/Cancer Hospital, Chinese Academy of Medical Sciences and Peking Union Medical College, Beijing, Beijing, China; 4 Department of Neurology, Peking Union Medical College Hospital, Chinese Academy of Medical Sciences & Peking Union Medical College, Beijing, China; 5 Department of Dermatology, Peking Union Medical College Hospital, Chinese Academy of Medical Sciences and Peking Union Medical College, State Key Laboratory of Complex Severe and Rare Diseases, National Clinical Research Center for Dermatologic and Immunologic Diseases, Beijing, China; 6 Department of Radiology, Peking Union Medical College Hospital, Chinese Academy of Medical Science & Peking Union Medical College, Beijing, China; 7 Department of Family Medicine & Division of General Internal Medicine, Department of Medicine, Peking Union Medical College Hospital, Chinese Academy of Medical Sciences & Peking Union Medical College, Beijing, China; 8 Division of Hematology, Department of Internal Medicine, Peking Union Medical College Hospital, Chinese Academy of Medical Sciences & Peking Union Medical College, Beijing, China

**Keywords:** cryoglobulinemia, immune-mediated injury, monoclonal gammopathy of clinical significance, multisystem inflammation, plasma cell disorders

## Abstract

Monoclonal gammopathy of clinical significance (MGCS) refers to disorders in which small B-cell or plasma-cell clones produce pathogenic monoclonal immunoglobulins that cause organ injury independent of tumor burden. Because the clinical spectrum is heterogeneous and diagnostic criteria remain evolving, MGCS is frequently underrecognized, particularly when organ manifestations precede detectable paraproteinemia. We report a 52-year-old man with multisystem manifestations including cutaneous xanthomatosis, inflammatory myopathy, cryoglobulinemia, and possible cardiac involvement. The patient initially presented with progressive polymyalgia, leukopenia, and yellow-brown cutaneous plaques. Immune abnormalities had been documented several years before detection of monoclonal protein. Laboratory evaluation revealed IgG-λ monoclonal gammopathy with type I cryoglobulinemia, complement consumption, and autoantibody positivity, indicating systemic immune activation. Bone marrow examination demonstrated only 1.4% λ-restricted clonal plasma cells. Skin biopsy confirmed xanthomatous infiltration, while muscle biopsy showed myopathic changes without amyloid deposition. Cardiac imaging demonstrated asymmetric septal hypertrophy with patchy late gadolinium enhancement. Treatment with bortezomib–cyclophosphamide–dexamethasone led to marked clinical improvement, including resolution of myalgia, regression of skin lesions, normalization of leukopenia, and disappearance of cryoglobulins, although low-level paraprotein persisted. This case highlights that immune-mediated organ injury may precede measurable clonal expansion in MGCS and supports the concept that pathogenic monoclonal immunoglobulins can act as immune effectors driving systemic inflammation.

## 1 Introduction

Monoclonal gammopathy of clinical significance (MGCS) refers to a condition in which a small, apparently indolent plasma-cell or B-cell clone produces monoclonal immunoglobulins that damage organs through immune-mediated or deposition mechanisms rather than tumor burden (1). Because diagnostic standards remain heterogeneous, MGCS is frequently under-recognized, particularly when organ involvement precedes detectable monoclonal protein (2). We report a MGCS patient with multi-organ involvement including skin, muscle and possibly heart—whose immune-mediated abnormalities preceded paraproteinemia by several years. The diverse paraneoplastic manifestations were incorporated into the spectrum of MGCS.

## 2 Case presentation

**Figure 1:**
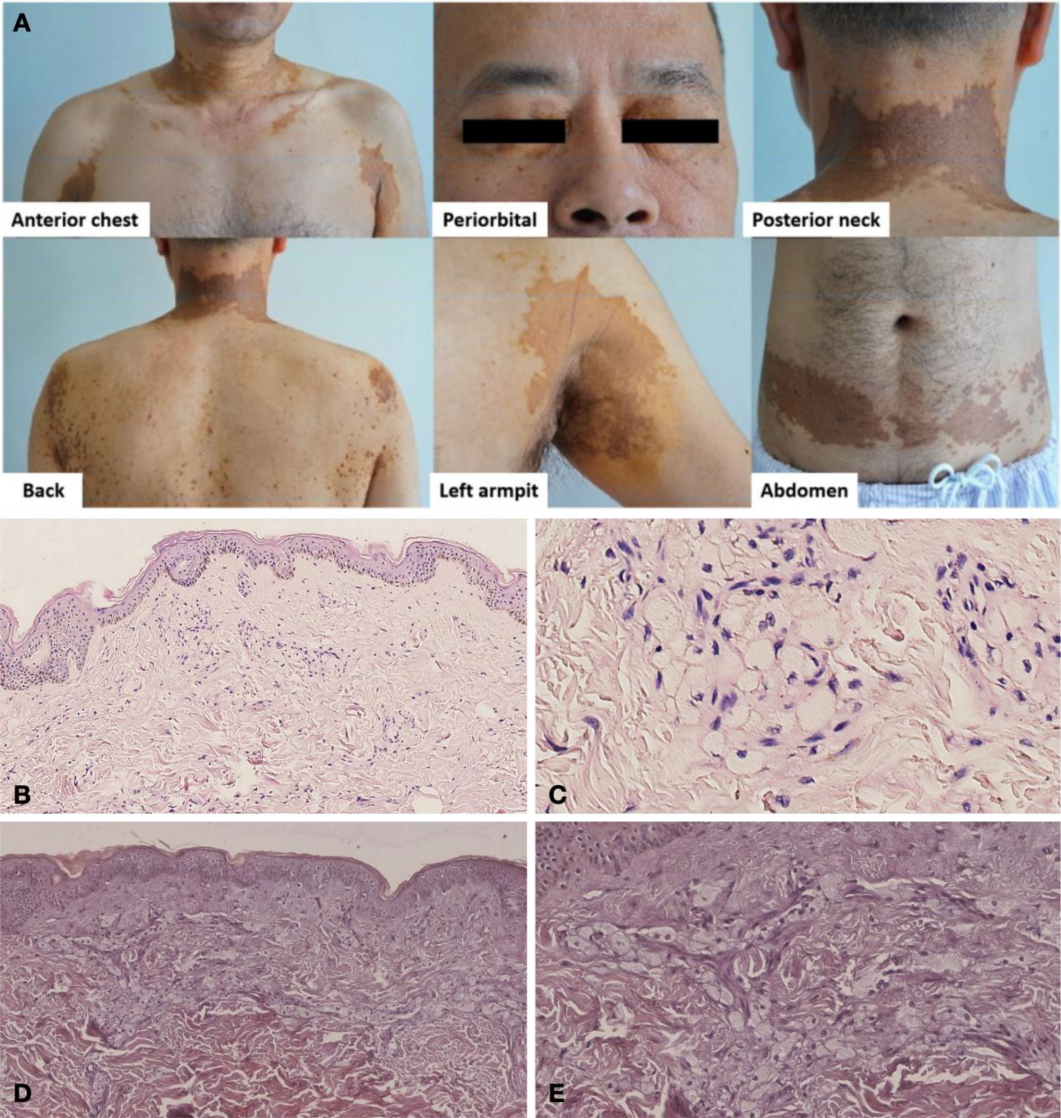
Representative cutaneous findings at presentation and histopathologic findings of skin biopsies. **(A)** Multiple, symmetric, flat, yellow-brown patches and plaques are visible on the anterior chest, posterior neck, back, abdomen, periorbital regions, and left axilla. The lesions are well-demarcated, slightly elevated with smooth surfaces, and some merged into large confluent plaques. **(B)** Increased basal layer pigmentation, with infiltration of foamy histiocytes in the superficial dermis and around small blood vessels. (hematoxylin & eosin, original magnification, ×100). **(C)** Foamy histiocytes with pale cytoplasm and eccentrically placed nuclei. (hematoxylin & eosin, original magnification, ×400). **(D)** The specimen stained negative for Congo red. (Original magnification, ×100). **(E)** The specimen stained negative for Congo red. (Original magnification, ×200).

A 52-year-old man was admitted to the ward of General Medicine for progressive polymyalgia, leukopenia, and yellow-brown cutaneous plaques (**Figure 1A**). He had a past history of exertional chest tightness attributed to coronary artery disease and underwent stenting in 2011. Since 2017, he had mild leukopenia (3.5–4 × 10^9^/L). In 2019, tissue biopsy of axillary plaques indicated xanthomatosis consisting of dermal foamy histiocytes. In 2021, cardiac MRI demonstrated asymmetric septal hypertrophy with preserved ejection fraction (66%). Over the following two years, leukopenia worsened, diffuse myalgia developed, and approximate 10kg weight loss were reported.

On admission in late 2023, asymmetric blood pressure was noted between arms (right 139/85 mmHg, left 115/70 mmHg). Physical examination revealed multiple yellow-brown plaques and hyperpigmented patches over the periorbital areas, neck, shoulders, axillae, and groins. Cardiac auscultation showed a grade 3/6 systolic murmur at the mitral and aortic areas with a diminished left radial pulse. The lungs were clear, the abdomen was soft and non-tender, and no focal neurological deficits were observed. Laboratory results showed white-blood-cell count 1.67 × 10^9^/L, hemoglobin 104 g/L, platelets 128 × 10^9^/L. Serum electrophoresis revealed an M-spike (IgG 12.4 g/L) affirming type I cryoglobulinemia (3.5%, 3.9 g/L) of IgG-λ component. The free κ/λ ratio was 0.178 (κ 23.0 mg/L, λ 129.6 mg/L). Urine immunofixation detected λ light chains. Tests related to hepatitis C virus infection or autoimmune diseases were negative. Creatine kinase remained normal, but post-exercise lactate rose to 3.7 mmol/L. Positive Coombs test; low complement C3 0.7 g/L, C4 0.021 g/L; ANA 1:80, anti-dsDNA 516 IU/mL incorporating ESR 77 mm/h, CRP 16.8 mg/L, IL-6 17.6 pg/mL suggested that immune system was activated. Bone marrow aspiration contained 1.4% λ-restricted clonal plasma cells with normal cytogenetics. PET–CT showed mild splenomegaly and diffuse marrow uptake without lytic lesions. Multiple myeloma, Waldenström macroglobulinemia, lymphoma, and myelodysplasia were excluded. Since hematological malignancies were excluded, MGCS presenting systemic involvement was suspected.

**Figure 2:**
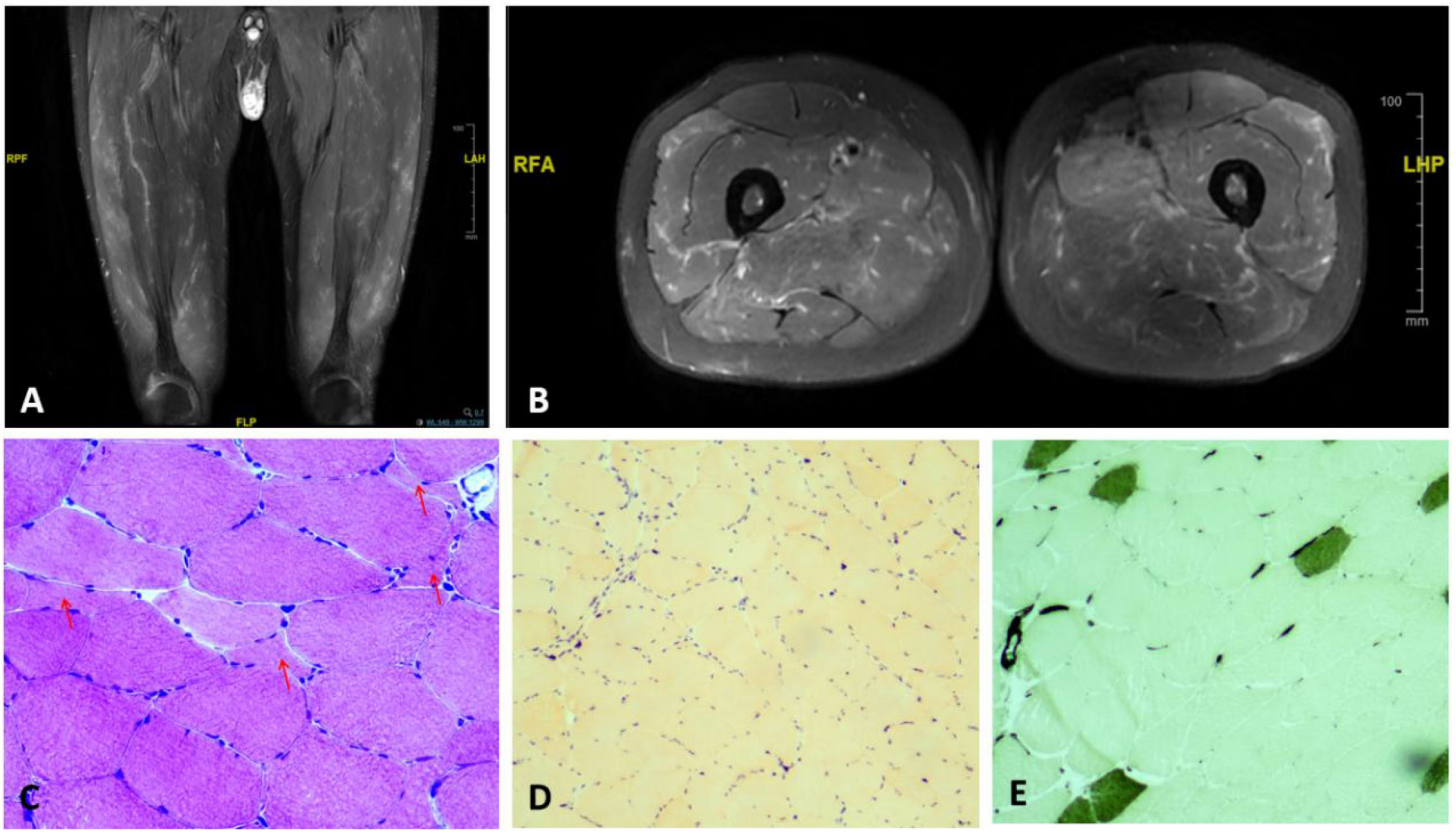
Thigh magnetic resonance imaging and muscle biopsy findings. **(A)** Axial T2-weighted fat-suppressed MRI of the thighs shows multiple patchy hyperintense signals within bilateral thigh and gluteal muscles, more prominent in the left anterior compartment. **(B)** Coronal MRI demonstrates heterogeneous marrow signal with multiple small ring-like lesions showing peripheral T2-hyperintensity with central hypointensity; cortical bone is intact. **(C)** Hematoxylin–eosin staining (400×) reveals marked variation in fiber size with scattered angular atrophic fibers (red arrows). **(D)** Congo red staining is negative for amyloid deposition. **(E)** ATPase staining (PH 4.3) demonstrates a predominance of type II muscle fibers.

Then, a skin biopsy from the posterior neck showed mild atrophy and thinning of spinous layer with basal layer hyperpigmentation (**Figure 1B**). In the superficial dermis, clusters of foamy histiocytes with pale cytoplasm and eccentrically placed nuclei were observed (**Figure 1C**), consistent with xanthomatous infiltration. No amyloid deposition or atypical histiocytic proliferation was identified (**Figures 1D, E**), confirming a diagnosis of cutaneous xanthomatosis. Muscle MRI revealed patchy T_2_ hyperintensity in the quadriceps and gluteal muscles (**Figures 2A, B**). Electromyography was normal. The tissue biopsy of quadriceps demonstrated myofibrillar disarray with scattered inflammatory infiltrates (**Figure 2C**), with negative Congo-red staining (**Figure 2D**), and negative BRAF V600E mutation by next generation sequencing (NGS). ATPase staining demonstrated a predominance of type II muscle fibers (**Figure 2E**). Besides, peripheral blood whole-exome sequencing did not detect relevant pathogenic mutations. The coexistence of cryoglobulinemia, complement consumption, and autoantibodies was consistent with an immune-complex process in which monoclonal immunoglobulins precipitate or bind self-antigens, activate complement, and induce tissue inflammation (3, 4).

**Figure 3:**
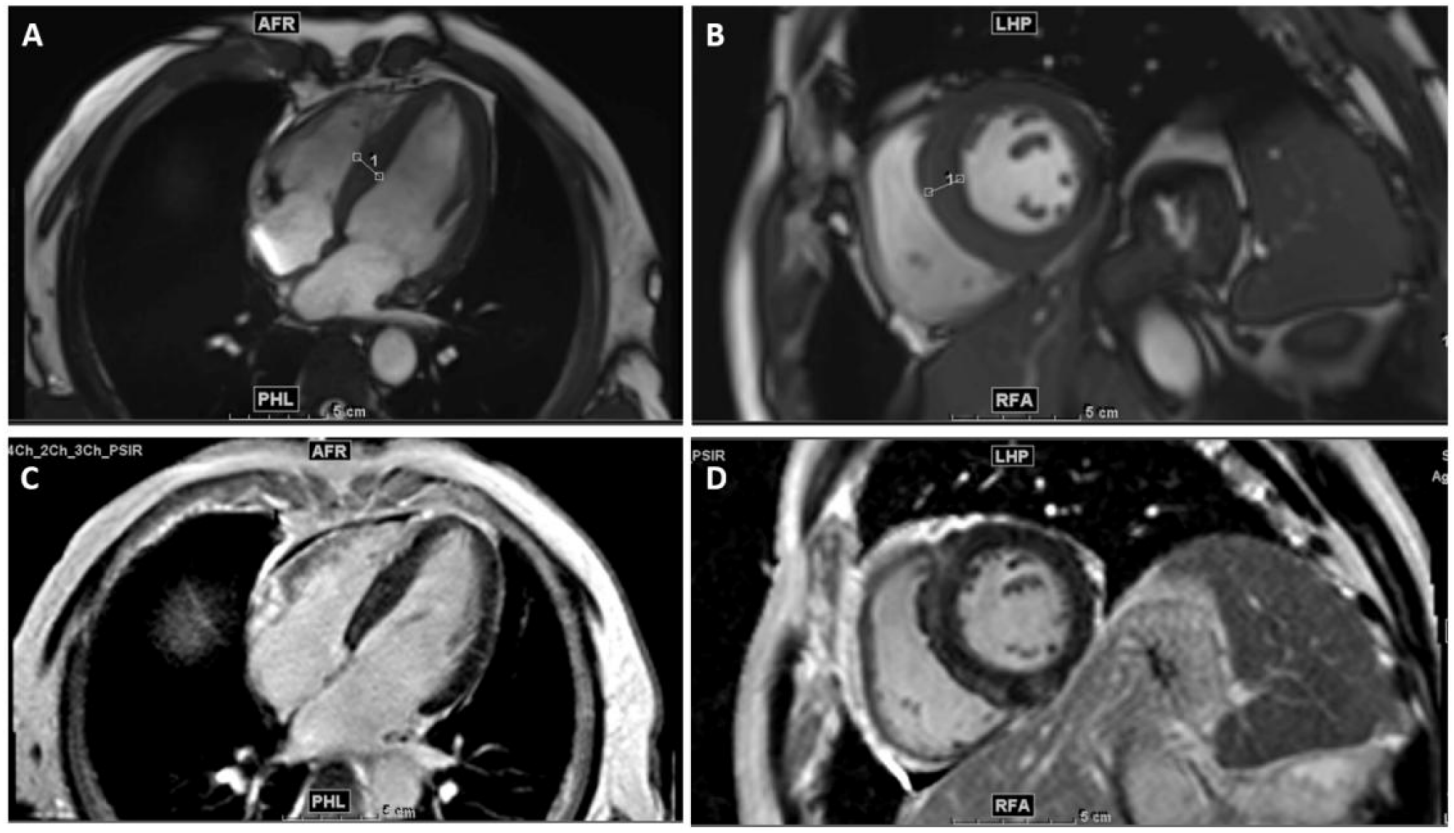
Cardiac MRI showing cine and PSIR late gadolinium enhancement (LGE) sequences. Cine bSSFP images **(A, B)** demonstrate asymmetric septal hypertrophy (white lines), whereas PSIR LGE images **(C, D)** show patchy mid-wall enhancement of the interventricular septum and inferolateral wall, consistent with an infiltrative cardiomyopathy pattern.

Echocardiography showed concentric hypertrophy with preserved systolic function and mild valvular regurgitation. Cardiac MRI revealed asymmetric septal thickening with late gadolinium enhancement (**Figure 3**). Given the patient’s coronary disease, cardiomyopathy was likely multifactorial. However, systemic immune activation and cryoglobulinemia raised the possibility that the monoclonal protein–related immune process contributed to secondary inflammatory remodeling (5). No amyloid deposition was detected.

A multidisciplinary team round comprising hematologists, cardiologists, dermatologists, neurologists, and pathologists concluded that cutaneous xanthomatosis, inflammatory myopathy, and cryoglobulinemia were associated with IgG-λ paraprotein, i.e. MGCS with multiorgan involvement (6). The gap between organ injury and monoclonal gammopathy suggests that immune reactions may have developed before plasma cell or B cell clones were detected (7). Differential diagnoses such as amyloidosis and histiocytosis were excluded by histopathological tests. In addition, systemic autoimmune diseases (including systemic lupus erythematosus and vasculitis) and infectious causes such as hepatitis C–associated cryoglobulinemia were considered but excluded based on clinical features and laboratory testing. Primary inflammatory myopathies were also unlikely given the absence of myonecrosis and normal electromyography.

**Figure 4:**
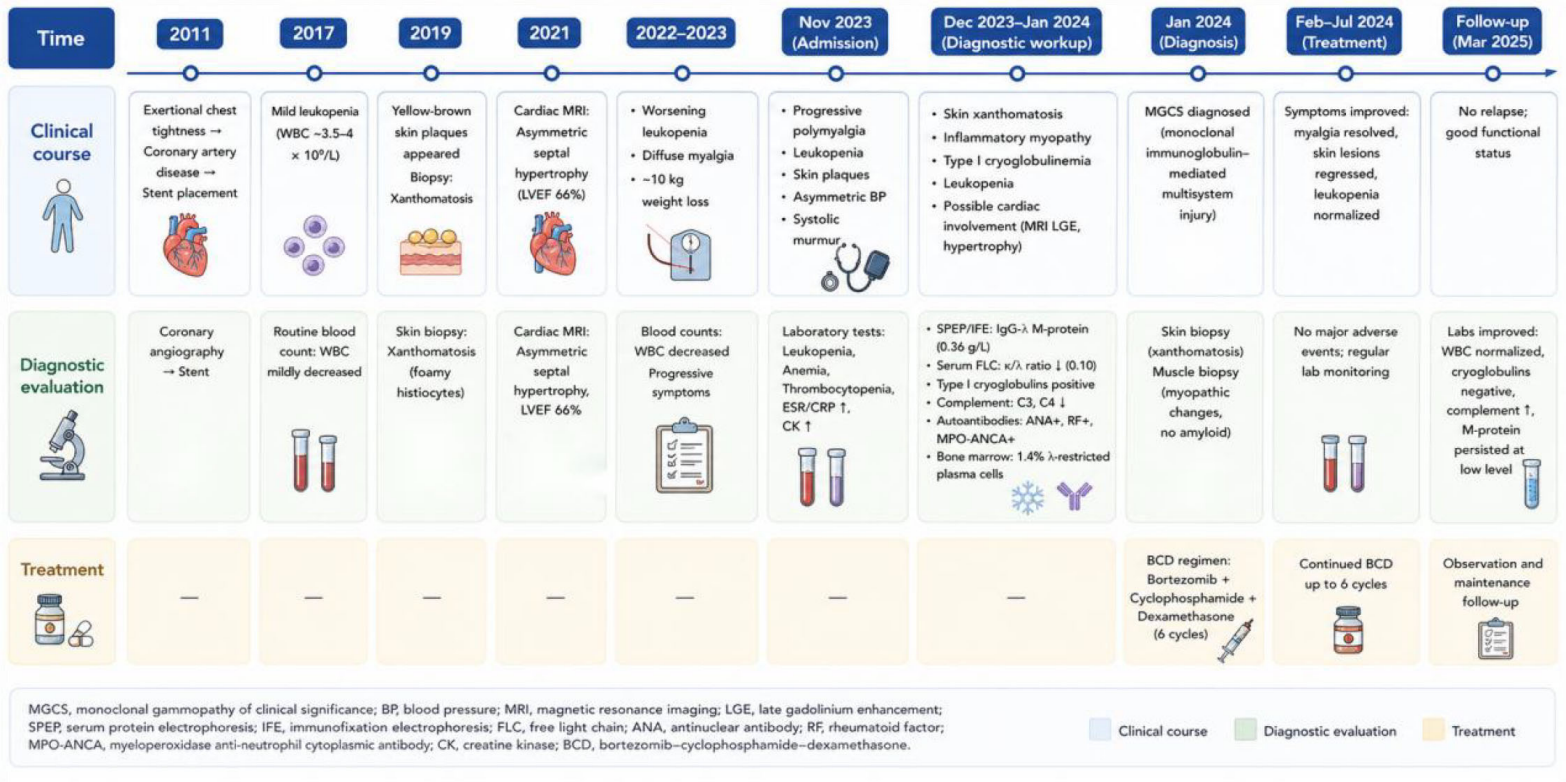
Timeline of clinical course, diagnostic evaluation, and treatment. The timeline summarizes the patient’s clinical progression from coronary artery disease and chronic leukopenia to cutaneous xanthomatosis, cardiac abnormalities, detection of IgG-λ monoclonal protein with type I cryoglobulinemia, MGCS diagnosis, bortezomib–cyclophosphamide–dexamethasone treatment, and 12-month follow-up.

A triplet regimen including Bortezomib–cyclophosphamide–dexamethasone (BCD) was administrated. The BCD regimen consisted of bortezomib (1.3 mg/m², subcutaneously, days 1, 8, 15, and 22), cyclophosphamide (300 mg/m², intravenously, days 1, 8, 15, and 22), and dexamethasone (20 mg, orally or intravenously, on the day of and the day after bortezomib administration), repeated every 28 days. After nine cycles, his serum M protein dropped to 0.79g/L with normal free light chain levels, achieving hematological very good partial response. The cryoglobulins became undetectable and skin lesions lightened. Myalgia resolved and exercise tolerance improved. Leukopenia improved (WBC 4.34 × 10^9^/L). The patient then received bortezomib maintenance every other week. Leukopenia recovered, cutaneous lesions regressed, and myalgia resolved, with marked improvement in laboratory parameters (**Table 1**). Cardiac echo demonstrated stable heart function at 12-month follow-up. The treatment was well tolerated, with no significant adverse events such as peripheral neuropathy or additional hematologic toxicity observed during therapy. The clinical course, diagnostic evaluation, and treatment response are summarized in **Figure 4**.

**Table 1:** Key laboratory parameters at baseline and after treatment.

Parameter	Baseline	Post-treatment
WBC (×10^9^/L)	1.67	4.34
HGB (g/L)	104	141
24h uMP (mg)	248	0
M protein (g/L)	12.4	0.79
sFLC-λ (mg/L)	129.6	28.8
Cryoglobulin(g/L)	3.9	0

WBC, white blood cell; HGB, hemoglobin; uMP, urine monoclonal paraprotein; sFLC, serum free light chain.

## 3 Discussion

The improvement of organ function developed before achieving complete hematologic response, a pattern previously noted in MGCS. The bortezomib-based regimen, as anti-plasma cell therapy, suppressed the production of paraproteins and downstream immune activation. Such hematologic–clinical discordance supports the understanding that immune toxicity more than tumor burden mostly drives organ damage (8, 9).

Three observations supported an immune-complex–mediated mechanism. First, monoclonal cryoglobulins can deposit in microvasculature and activate complement, leading to inflammation and cytopenias (3). Second, the long-term gap between cutaneous and muscular symptoms and the later M-protein detection suggested that autoantibody-like paraprotein activity may have preceded overt paraproteinemia (10, 11). Third, autoantibody positivity and elevated cytokines indicated persistent immune stimulation. While these associations cannot confirm causation, they align with current models of MGCS as an immune-toxic rather than proliferative disorder (1).

Cutaneous xanthomatosis and inflammatory myopathy coexisting in MGCS are exceptionally uncommon. Xanthomatous lesions may arise from macrophage lipid accumulation promoted by chronic immunoglobulin exposure (12). The muscle pathology resembles sporadic late-onset nemaline myopathy (SLONM) associated with IgG-λ paraproteins; both improve with clone-directed therapy (13). The patient’s hypertrophic cardiomyopathy likely reflected a combination of ischemic remodeling and possible immune modulation. Although a direct causal link to MGCS cannot be proven, the coexistence of cryoglobulinemia and complement activation could plausibly exacerbate existing myocardial changes (2). MGCS in this context is best regarded as a disease modifier rather than a unifying etiology.

The present case also highlights the growing conceptual overlap between plasma cell neoplasms and paraneoplastic syndromes recognized in the 5th edition of the World Health Organization Classification of Haematolymphoid Tumors (WHO-HAEM5) (14). In this revision, plasma cell disorders are defined not only by tumor burden but also by pathogenic monoclonal immunoglobulin–mediated or cytokine-mediated injury.

Our patient shares mechanistic similarities with these disorders. This pattern is compatible with an immune-paraneoplastic mechanism in which monoclonal protein acts as a pathogenic antibody or cryoglobulin, forming immune complexes and inducing inflammation across multiple organs. Conceptually, this case lies within the expanding spectrum of MGCS, bridging monoclonal gammopathies and paraneoplastic plasma cell syndromes (14). It underscores that small plasma cell clones can function as immune effectors whose products cause clinically significant paraneoplastic injury.

From a diagnostic perspective, this case highlights the need for coordinated hematologic, immunologic, and organ-specific evaluation in unexplained multisystem disease. MGCS should be considered in patients with atypical xanthomatous dermatoses or inflammatory myopathy even when monoclonal protein levels are minimal. Repeated testing may be required, as M-protein detection can lag behind clinical manifestations (15). Early bone-marrow examination and clone-directed therapy may prevent irreversible injury.

The patient’s favorable and durable response to BCD and maintenance underscores the benefit of plasma-cell–directed regimens in non-renal MGCS. Persistent low-level paraprotein does not necessarily predict relapse; follow-up should emphasize organ and immunologic parameters. Progression risk to overt myeloma is low (about 1% per year) but warrants surveillance (5). The role of autologous stem-cell transplantation remains uncertain and should be reserved for refractory cases (6).

Future studies are needed to identify biomarkers that may predict disease activity or organ flares in MGCS, including dynamic changes in monoclonal protein levels, complement consumption, and inflammatory cytokines.

This case has several strengths, including comprehensive multisystem evaluation and a clear demonstration of hematologic–clinical discordance, as well as a favorable response to clone-directed therapy supporting the diagnosis. However, it is limited by the single-patient nature and the inability to establish causality, particularly regarding cardiac involvement in the context of pre-existing coronary artery disease.

In summary, MGCS with cutaneous, muscular, and possible cardiac manifestations was diagnosed after a prolonged prodrome during which immune injury preceded paraproteinemia. It demonstrated that a small clonal population can provoke systemic inflammation through cryoglobulin and immune-complex activity (4). The case broadens current understanding of MGCS pathophysiology and emphasizes that immune injury may manifest years before measurable monoclonal protein. In patients with concurrent cardiovascular disease, MGCS should be viewed as a potential disease modifier rather than the sole cause of cardiac pathology. Recognition of such presentations will facilitate earlier diagnosis and individualized management.

## 4 Patient perspective

The patient reported a long and challenging diagnostic journey spanning nearly 10 years, during which symptoms progressively worsened without a clear diagnosis. Following treatment, he experienced significant relief of muscle pain and improved physical endurance. The regression of skin lesions and stabilization of laboratory parameters provided reassurance and improved his confidence in disease management.

## Data Availability

The original contributions presented in the study are included in the article/supplementary material. Further inquiries can be directed to the corresponding authors.
